# Interpretable bioimpedance-based modeling of body composition and metabolic indices related to physical function in maintenance hemodialysis patients

**DOI:** 10.3389/fnut.2026.1849618

**Published:** 2026-07-22

**Authors:** Jiahui Ding, Yanjuan Teng

**Affiliations:** Department of Nephrology, Shanghai Sixth People’s Hospital Affiliated to Shanghai Jiao Tong University School of Medicine, Shanghai, China

**Keywords:** bioelectrical impedance analysis, body composition, ensemble regression models, maintenance hemodialysis (MHD), physical function, SHAP analysis

## Abstract

**Objective:**

In maintenance hemodialysis (MHD) patients, muscle wasting and altered body composition are major determinants of reduced physical function. This study aimed to identify the key body composition features associated with four physical-function-related indices—Basal Metabolic Rate (BMR), Fat-Free Mass (FFM), Skeletal Muscle Mass (SMM), and Percentage of Body Fat (PBF)—and to evaluate whether ensemble regression models can estimate these indices from routine bioelectrical impedance analysis (BIA) data.

**Methods:**

Ensemble regression models were constructed using BIA-derived indicators to estimate BMR, FFM, SMM, and PBF. The SHAP algorithm was employed to assess feature importance. Individual regression models (LR, DT, SVR, GBR, Adaboost, and KNN) were compared with ensemble models (RF, ET, and LGBM) using *R*^2^, MAE, MSE, and RMSE.

**Results:**

SHAP analysis identified Total Body Water (TBW) and height as the most significant predictors across all four indices, followed by Intracellular Water (ICW), Extracellular Water (ECW), minerals, and body weight. Ensemble models consistently outperformed individual models. LGBM achieved the highest performance (e.g., *R*^2^ = 0.99, RMSE = 0.11 for BMR), markedly surpassing individual models such as DT (*R*^2^ = 0.89, RMSE = 0.39). Similar trends were observed for SMM, FFM, and PBF.

**Conclusion:**

Ensemble learning models, particularly LGBM, demonstrated high apparent accuracy in estimating the four indices. SHAP analysis consistently identified TBW and height as dominant contributors. We caution that this predictive strength partly reflects expected physiological relationships between body water, body size, and these indices, rather than novel clinical insights. The practical value lies in providing an interpretable body composition profile that, if externally validated, may help identify MHD patients at risk of muscle wasting and poor functional status. Because this study used only internal validation and had no independent external validation cohort, clinical application requires validation against directly measured functional outcomes in larger, multicenter, longitudinal cohorts, with body composition standardized to a fixed point in the dialysis cycle.

## 1 Introduction

Maintenance hemodialysis (MHD) is the most widely used form of renal replacement therapy for patients with end-stage renal disease (ESRD), and for the large proportion of patients who are not candidates for kidney transplantation it constitutes the principal life-sustaining treatment. However, long-term complications of MHD, such as protein-energy wasting and skeletal muscle atrophy, significantly impair patients’ physical function and exercise capacity, leading to decreased functional independence and adverse outcomes (1–3). Studies have shown that there is a complex interplay between body composition abnormalities in MHD patients (such as increased body fat percentage, reduced muscle mass, and imbalanced extracellular water ratio) and physical function and activity capacity. However, traditional linear regression or univariate analysis is insufficient to capture the dynamic associations (4, 5).

In recent years, the recent advances of machine learning (ML) in the medical field has provided useful tools for interpreting complex physiological data. Its advantage lies in the ability to identify potential patterns among high-dimensional features through nonlinear modeling and to optimize predictive performance through feature engineering. For example, algorithms such as random forest (RF), feedforward neural networks, and independent component analysis can effectively handle multi-collinear data and have demonstrated high precision in ESRD risk stratification (6–8). Meanwhile, clustering analysis (such as K-means or hierarchical clustering) can reveal heterogeneous patient subgroups, providing targets for precision interventions (9, 10). However, for MHD patients, existing ML studies have largely focused on biochemical indicators or survival prediction, without fully exploring the interpretable associations between multimodal body composition data (such as mineral content, trunk-to-limb muscle ratio, and intracellular/extracellular water ratio) and physical-function-related indices. Moreover, how to transparently characterize the model-based associations of key body composition variables remains insufficiently explored.

To address these gaps, the present study uses interpretable machine learning to examine how routinely measured body composition relates to four indices that are clinically relevant to physical function in MHD patients: Basal Metabolic Rate (BMR), Fat-Free Mass (FFM), Skeletal Muscle Mass (SMM), and Percentage of Body Fat (PBF). We deliberately treat these four indices as markers of muscle mass, resting energy metabolism, and fat distribution—the body composition substrate of physical functional capacity—rather than as direct measures of physical activity behavior, which would require accelerometry or validated questionnaires. Clinically, low SMM and FFM and unfavorable PBF are established correlates of sarcopenia, frailty, and protein-energy wasting in dialysis patients, conditions that predict falls, hospitalization, and mortality; an interpretable, impedance-based description of these indices could therefore help identify which patients to prioritize for nutritional and exercise intervention. We constructed ensemble regression models from comprehensive body composition data and applied SHAP (SHapley Additive exPlanations) analysis to quantify, in a transparent way, which body composition parameters contribute most to each index. We emphasize that SHAP attributions describe associations within the model and should not be read as causal effects. Our aim is to provide a transparent, reproducible framework and to identify the body composition variables most strongly linked to these functionally relevant indices in MHD patients.

## 2 Methods

### 2.1 Dataset information

In accordance with KDIGO 2024 guidelines, we identified adult (≥18 years) patients with end-stage kidney disease (ESKD, ICD-10 code N18.6) who had been receiving maintenance hemodialysis (MHD) for at least three consecutive months by 1 January 2024. Among these patients, those who remained on MHD between 1 January and 31 December 2024 and met the additional criteria listed in Table 1 were consecutively enrolled (*n* = 447). The relatively strict eligibility criteria, including the exclusion of major comorbidities such as diabetes and cardiovascular disease and the restricted age, BMI, and body-fat ranges, were applied deliberately to obtain a comparatively homogeneous cohort in which body-composition relationships could be characterized with reduced confounding from comorbidity-related metabolic and fluid disturbances. We acknowledge, however, that diabetes and cardiovascular disease are among the most common comorbidities in the MHD population, and that their exclusion necessarily reduces the representativeness and external generalizability of the cohort; this trade-off is examined further in the Discussion, and the resulting need for validation in unselected, multi-morbid MHD populations is highlighted as a key direction for future work. All patients were treated at a single tertiary center. For each patient, the model input comprised demographic variables (age, sex, height, body weight) together with the full set of parameters produced by a multi-frequency segmental bioelectrical impedance analysis (BIA) body composition analyzer (InBody 770; InBody Co., Ltd., Seoul, Republic of Korea). These included whole-body and segmental water compartments (total body water, intracellular water, and extracellular water for the trunk and each limb), fat-free mass, skeletal muscle mass, body cell mass (BCM), total mineral content, body fat mass, and segmental impedance measured at multiple frequencies (50 kHz and 500 kHz). It is important to note that all candidate features were derived from demographics and a single BIA device; no laboratory, dialysis-prescription, comorbidity, dietary, or directly measured physical-activity variables were available, which constrains the clinical scope of the models. The complete list of all exported variables, their units, and their role in the models (input, target, or not used) is provided in Supplementary Table S1; the models used 120 input features (116 continuous bioimpedance/anthropometric variables plus sex and a >65 vs. ≤65 age indicator, one-hot encoded), with BMR, FFM, SMM and PBF as prediction targets. No formal *a priori* sample-size or power calculation was performed, because the study used consecutive, complete enrollment of all eligible MHD patients within a fixed 12-month window rather than sampling to detect a pre-specified effect size. *Post hoc*, this sample is adequate for the intended descriptive modeling: after the 8:2 split, the training set and the held-out test set provide a sufficient number of observations per predictor for stable estimation with regularized and ensemble regressors, and 5-fold cross-validation was used during hyperparameter tuning to limit overfitting. We nonetheless acknowledge that this is a single-center consecutive sample, and that larger, multicenter cohorts would be required both for formal external validation and for any future analyses involving pre-specified hypothesis testing or minimum-detectable-effect calculations (see Table 2).

**Table 1:** The baseline information included in the research sample.

Item	Inclusion criteria	Exclusion criteria
Age	18–65 years old	Less than 18 years old or more than 65 years old
Gender	Male or female	—
Weight	45–120 kg	Less than 45 kg or more than 120 kg
Height	155–190 cm	Less than 155 cm or more than 190 cm
BMI	19–28	Less than 19 or more than 28
Body fat percentage	15–35%	Less than 15% or more than 35%
Health status	No major chronic diseases (e.g., cardiovascular diseases, diabetes)	Presence of major chronic diseases (e.g., cardiovascular diseases, diabetes)
Pregnancy or lactation	—	Pregnant or lactating women
Recent study participation	—	Participated in other similar studies within the past 3 months
Willingness to participate	Willing to sign the informed consent form and able to complete the study procedures and cooperate with the study	Unable to complete the study procedures or unwilling to cooperate with the study

**Table 2:** Baseline demographic, clinical and body composition characteristics of the study cohort (*n* = 447).

Characteristic	Overall (N = 447)	Male (n = 208)	Female (n = 239)
Sex, *n* (%)	—	208 (46.5)	239 (53.5)
Age (years)	50 ± 16	51 ± 16	48 ± 16
	49 (36–62)	54 (39–65)	45 (36–59)
Height (cm)	165.9 ± 8.5	172.5 ± 6.8	160.1 ± 4.8
	165.0 (160.0–170.0)	171.0 (168.0–176.0)	160.0 (157.5–163.0)
Weight (kg)	64.1 ± 13.9	71.7 ± 15.3	57.6 ± 7.9
	60.4 (55.6–69.2)	67.8 (59.2–81.1)	58.0 (52.0–62.1)
BMI (kg/m^2^)	23.2 ± 3.7	24.0 ± 4.3	22.4 ± 2.9
	22.5 (20.7–25.0)	23.1 (20.7–26.9)	21.9 (20.7–24.3)
Total body water, TBW (L)	35.1 ± 7.5	40.9 ± 6.8	30.0 ± 3.1
	32.8 (29.8–40.1)	40.8 (35.7–45.4)	30.0 (28.1–31.8)
Intracellular water, ICW (L)	21.4 ± 4.6	25.0 ± 4.2	18.4 ± 1.9
	20.2 (18.3–24.7)	24.9 (22.1–26.9)	18.4 (17.1–19.2)
Extracellular water, ECW (L)	13.7 ± 3.0	16.0 ± 2.8	11.7 ± 1.3
	12.7 (11.5–15.4)	15.8 (13.9–17.7)	11.6 (11.0–12.3)
Fat-free mass, FFM (kg)	47.7 ± 10.1	55.5 ± 9.2	40.9 ± 4.2
	44.6 (40.5–54.5)	55.2 (48.4–61.3)	40.8 (38.2–43.2)
Skeletal muscle mass, SMM (kg)	26.0 ± 6.0	30.6 ± 5.4	21.9 ± 2.5
	24.3 (21.9–30.2)	30.5 (26.8–33.1)	22.0 (20.4–23.1)
Basal metabolic rate, BMR (kcal/day)	1,400 ± 218	1,569 ± 199	1,253 ± 91
	1,333 (1246–1,546)	1,562 (1416–1,694)	1,251 (1195–1,304)
Percent body fat, PBF (%)	25.2 ± 8.6	21.5 ± 8.5	28.4 ± 7.3
	25.7 (19.2–31.6)	21.9 (14.7–28.0)	28.4 (24.1–34.2)
Body fat mass, BFM (kg)	16.5 ± 7.5	16.2 ± 9.0	16.7 ± 6.0
	15.6 (10.8–21.2)	14.5 (8.9–22.2)	16.1 (12.8–20.9)
Minerals (kg)	3.31 ± 0.69	3.79 ± 0.70	2.89 ± 0.30
	3.12 (2.88–3.66)	3.67 (3.31–4.20)	2.89 (2.74–3.03)

### 2.2 Data preprocessing

Data preprocessing was accomplished using the Python programming language and the pandas library. The dataset was loaded into a pandas DataFrame. Within the eligible age window of 18–65 years (Table 1), the continuous age variable was additionally dichotomized for encoding into “≤40 years” and “>40 years”; the 40-year cut-point was chosen *a priori* as it approximated the cohort median and produced balanced subgroups. This 40-year encoding threshold is used only as a model input and is distinct from the 65-year upper eligibility limit reported in Table 1. Missing values were handled by mean imputation (scikit-learn SimpleImputer, strategy = “mean”); in the analyzed dataset none of the retained model-input variables contained missing values, so no imputation was applied in practice. No automated feature-selection step was applied to the predictor set; instead, the full set of demographic and bioimpedance features was retained, and feature relevance was assessed *post hoc* using SHAP values (Section 2.3). Columns were divided into discrete variables (“Gender” and “Age”) and continuous variables. Discrete variables were one-hot encoded using the pd.get_dummies function, and the encoded specific columns were converted to integer data types. Continuous variables were processed using *Z*-score normalization, which involved subtracting the mean and dividing by the standard deviation for each numerical column. Finally, the preprocessed continuous and discrete variable DataFrames were merged along the column axis to serve as the final model input.

### 2.3 Construction of ensemble regression models

The dataset was split into training and testing sets in an 8:2 ratio, with a fixed random state. For each target index, the target variable itself was excluded from the predictor set. Variables that were mathematically or device-derived components of the target were also carefully considered to reduce circular prediction. Specifically, when SMM was the target, FFM and BCM were removed; when FFM was the target, SMM, BCM, and FFM-equivalent segmental lean-mass values were removed; when PBF was the target, body fat mass (BFM), segmental fat-mass terms, and BMI were removed; and when BMR was the target, any device-reported metabolic-rate surrogate was removed. We nonetheless emphasize that, because every predictor and every target originate from the same single bioimpedance device and are computed by the manufacturer from a shared pool of raw resistance/reactance measurements, residual structural and algorithmic overlap between predictors and targets cannot be fully eliminated; the reported errors should therefore be read as internal descriptive performance rather than as independent clinical prediction. An ensemble-based stacking regressor method was employed, integrating the predictive capabilities of multiple base models, including Linear Regression (LR), Decision Tree Regression (DT), Random Forest Regression (RF), Support Vector Regression (SVR), Gradient Boosting Regression (GBR), AdaBoost Regression (ADA), K-Nearest Neighbors Regression (KNN), Extra Trees Regression (ET), and LightGBM Regression (LGBM). Hyperparameter tuning was performed using GridSearchCV with 5-fold cross-validation, and the optimal parameters were selected based on the *R*^2^ score. In the stacking ensemble, only the meta-learner (final_estimator, a Random Forest) was tuned by GridSearchCV; the search grid was *n*_estimators ∈ {10, 20, 50}, max_depth ∈ {5, 10, 20}, and min_samples_split ∈ {2, 5, 10}. The eight base learners were used with fixed configurations (Supplementary Table S2B); in particular, LightGBM (version 4.6.0) was configured with n_estimators = 100, learning_rate = 0.1, num_leaves = 31, and max_depth = −1 (no limit). The meta-learner hyperparameters selected for each target index are reported in Supplementary Table S2A. The random_state was fixed at 42 for all stochastic components to ensure reproducibility, and *Z*-score normalization was fitted on the training folds only and applied to the validation/test data to prevent leakage. The performance of the stacking model and each base model was evaluated using *R*^2^ score, Mean Absolute Error (MAE), Mean Squared Error (MSE), and Root Mean Squared Error (RMSE). SHAP (SHapley Additive exPlanations) values were calculated to interpret the contribution of each feature to model predictions, and summary and dependence plots were generated for visualization. Additionally, comparison plots of actual versus predicted values and bar charts of base model performance with numerical labels were created to assess model performance and prediction outcomes. All analyses were implemented in Python (version 3.10.20). Data loading and preprocessing used pandas (version 2.3.3) and NumPy (version 2.2.6); model construction, the stacking regressor, GridSearchCV-based hyperparameter tuning with 5-fold cross-validation, *Z*-score (StandardScaler) normalization, and the clustering algorithms used scikit-learn (version 1.7.2); gradient-boosted tree models used LightGBM (version 4.6.0); feature attribution used the SHAP package (version 0.42.0); and all figures were generated with Matplotlib (version 3.10.9). Exact package versions are reported to support computational reproducibility.

### 2.4 Construction of clustering models

In this study, we analyzed a dataset containing multiple physiological variables using various clustering algorithms. First, the data were standardized using the StandardScaler function to ensure that all features had a mean of 0 and a standard deviation of 1. Subsequently, five clustering algorithms—KMeans, Agglomerative Clustering, Gaussian Mixture Model (GMM), Spectral Clustering, and BIRCH—were evaluated within a range of 2 to 10 clusters. To quantify clustering performance, an evaluation function named evaluate_clustering was defined, using three metrics: Silhouette Score, Calinski-Harabasz index, and Davies-Bouldin index, to measure the separation, cohesion, and overall performance of the clusters. Line plots of the three evaluation metrics as a function of the number of clusters were drawn to intuitively compare the performance of different algorithms. Moreover, the optimal parameter combinations for each algorithm were selected based on the silhouette coefficient, and t-SNE was used to project the data into a two-dimensional space for visualization.

### 2.5 Association analysis method

Canonical Correlation Analysis (CCA) was utilized in this study to analyze the relationships between target indices and their associated top features. Initially, a CCA model with one component was initialized and fitted to the training data, followed by transforming the data into the canonical space to obtain canonical variables. The canonical correlation coefficients were calculated for both the training and testing sets to assess the strength of the relationships. The distribution of canonical variables was visualized using scatter plots, and the top 10 most influential features were identified by extracting and ranking the feature weights from the CCA model. These features were further visualized using horizontal bar charts.

## 3 Results

### 3.1 Identification of factors associated with body composition and metabolic indices related to physical function based on ensemble regression models

**Figure 1:**
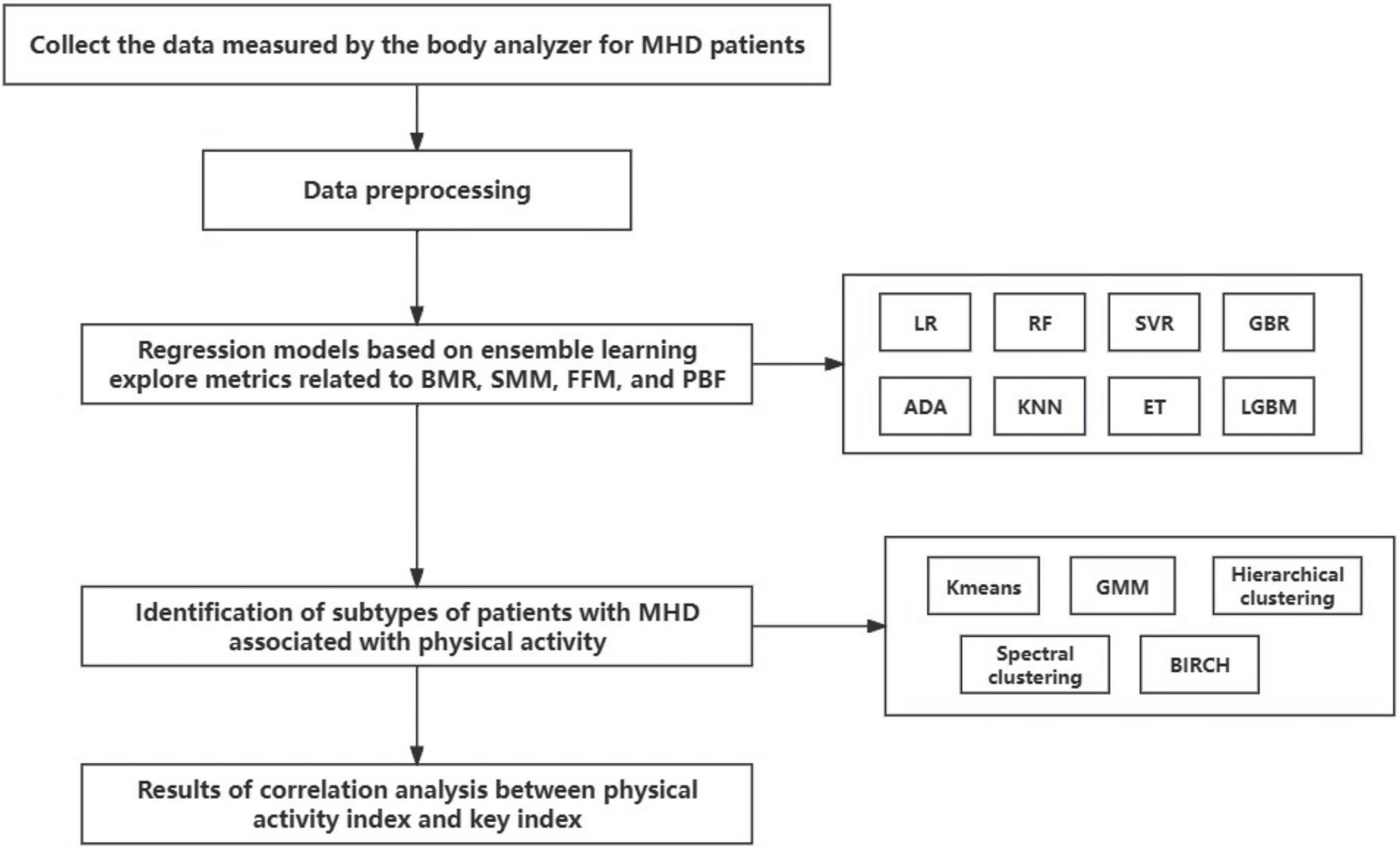
The technical roadmap for this paper.

**Figure 2:**
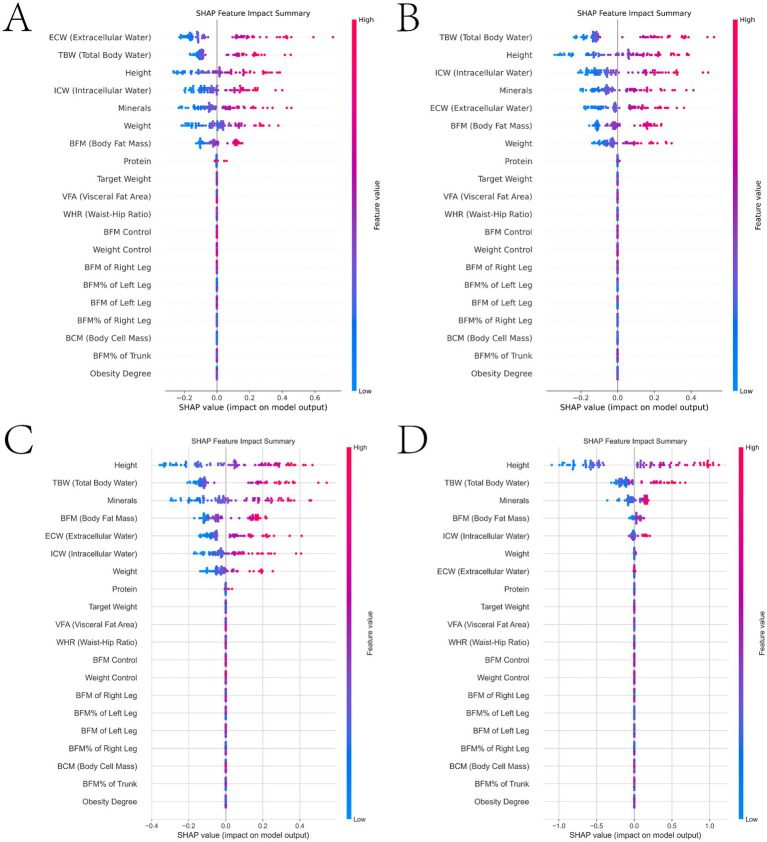
SHAP-based feature importance for the four indices (**(A)** BMR; **(B)** FFM; **(C)** SMM; **(D)** PBF). Larger absolute SHAP-values (*x*-axis) indicate greater influence of a feature on the model output.

In this study, an ensemble model constructed based on multiple regression algorithms was employed to estimate four physical-function-related body composition and metabolic indices (BMR, FFM, SMM, and PBF) using various indices from a body composition analyzer (Figure 1). Subsequently, the SHAP algorithm was utilized to calculate the feature importance assessment results (Figures 2A–D). In the analysis of the BMR indicator, ECW, TBW, and height were identified as the most important features influencing model prediction. Additionally, ICW, minerals, body weight, and BFM also made significant contributions to the model prediction. For the FFM indicator, TBW and height were the most important features, exhibiting the highest SHAP values. ICW, minerals, body weight, and BFM likewise had a substantial impact on the model prediction. In the analysis of the SMM indicator, height and TBW were the most important features, showing the highest SHAP values. Minerals, BFM, ECW, and ICW also significantly influenced the model prediction. For the PBF indicator, height and TBW were again among the most important features after excluding directly related fat-mass variables. Other retained features, including minerals, ECW, and ICW, also contributed to the model output. Through SHAP model analysis of the four different indicators (BMR, FFM, SMM, and PBF), we found that TBW and height were the most important features influencing model prediction, with the highest SHAP values.

**Figure 3:**
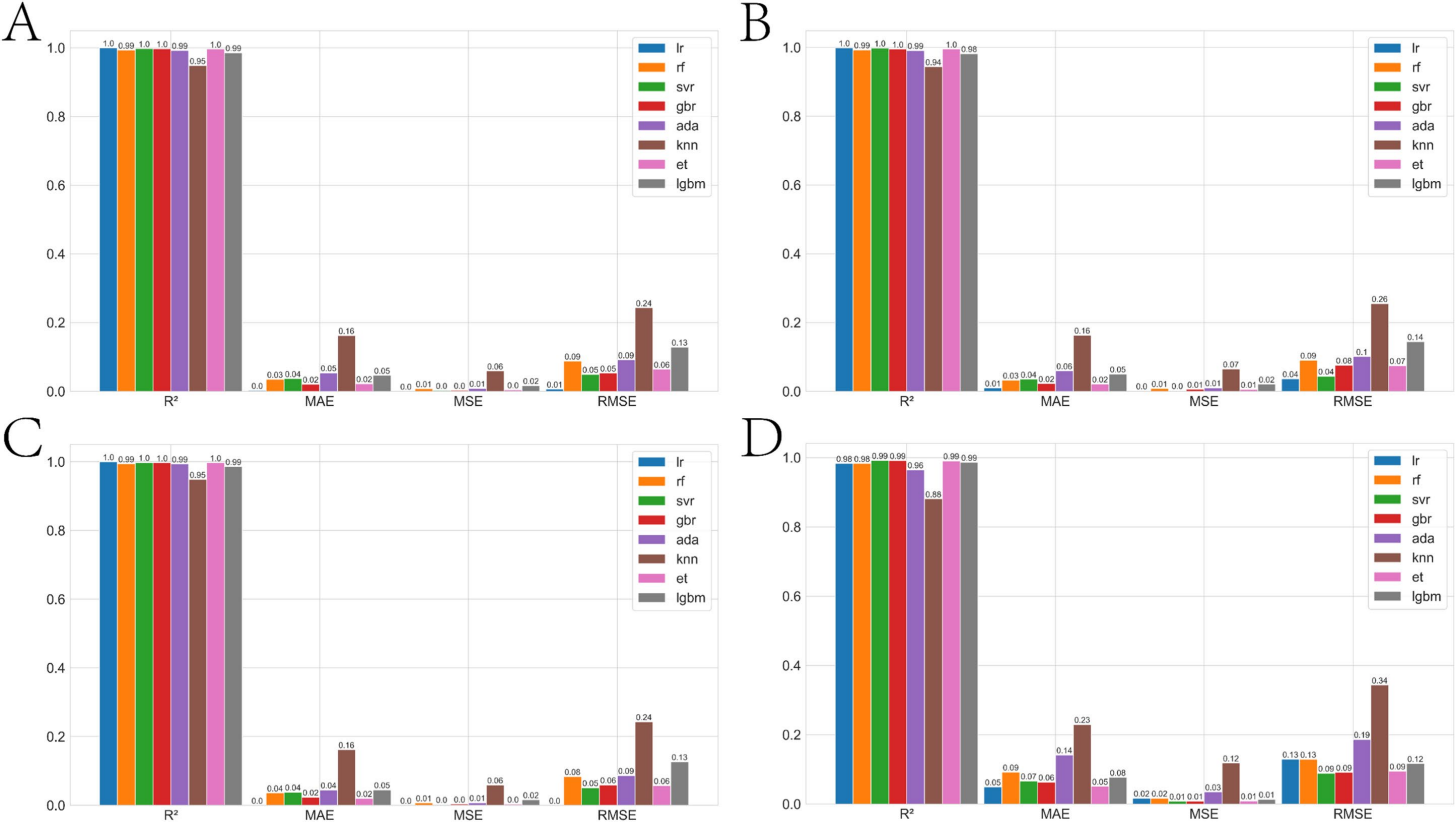
Comparison of regression performance. Panels **(A–D)** compare the regression performance of individual models (such as linear regression, decision tree, support vector machine, etc.) and ensemble learning models in predicting body composition and metabolic indices related to physical function (BMR, SMM, FFM, and PBF). The *x*-axis represents different models, and the *y*-axis represents regression performance metrics (such as *R*^2^, MAE, MSE, and RMSE). The figure illustrates the performance of each model on the same dataset to evaluate the advantages of ensemble learning models over individual models.

Figures 3A–D provide a detailed comparison of the regression performance of different models in predicting the four body composition and metabolic indices related to physical function (BMR, SMM, FFM, and PBF). These models included individual models such as LR, DT, SVR, GBR, Adaboost, and KNN, as well as ensemble learning models such as RF, ET, and LGBM. In the prediction of all four indicators, ensemble learning models generally exhibited superior performance compared to individual models. Specifically, the RF, LGBM, and ET models performed the best in all regression performance metrics, including *R*^2^, MAE, MSE, and RMSE. For example, in estimating BMR, the LGBM model reached an *R*^2^ value of 0.99. This high value should be interpreted cautiously, because BMR and the retained predictors were derived from the same BIA system and are therefore partly structurally related. Similarly, these ensemble models showed similar advantages in predicting SMM, FFM, and PBF. In contrast, individual models such as DT performed worse than ensemble models in all indicators. For instance, in predicting BMR, the *R*^2^ value of the DT model was 0.89, with an MAE of 0.15, MSE of 0.15, and RMSE of 0.39, all of which were significantly lower than those of ensemble models. This trend was also observed in the prediction of SMM, FFM, and PBF, where the DT model scored lower than ensemble models in all these indicators. Ensemble models showed lower prediction errors for these four body composition and metabolic indices related to physical function within this dataset. However, because the predictors and outcomes were derived from the same BIA system, these performance metrics should be interpreted as internal descriptive performance rather than evidence of independent clinical prediction.

### 3.2 Exploratory identification of body-composition-based clusters in MHD patients

**Figure 4:**
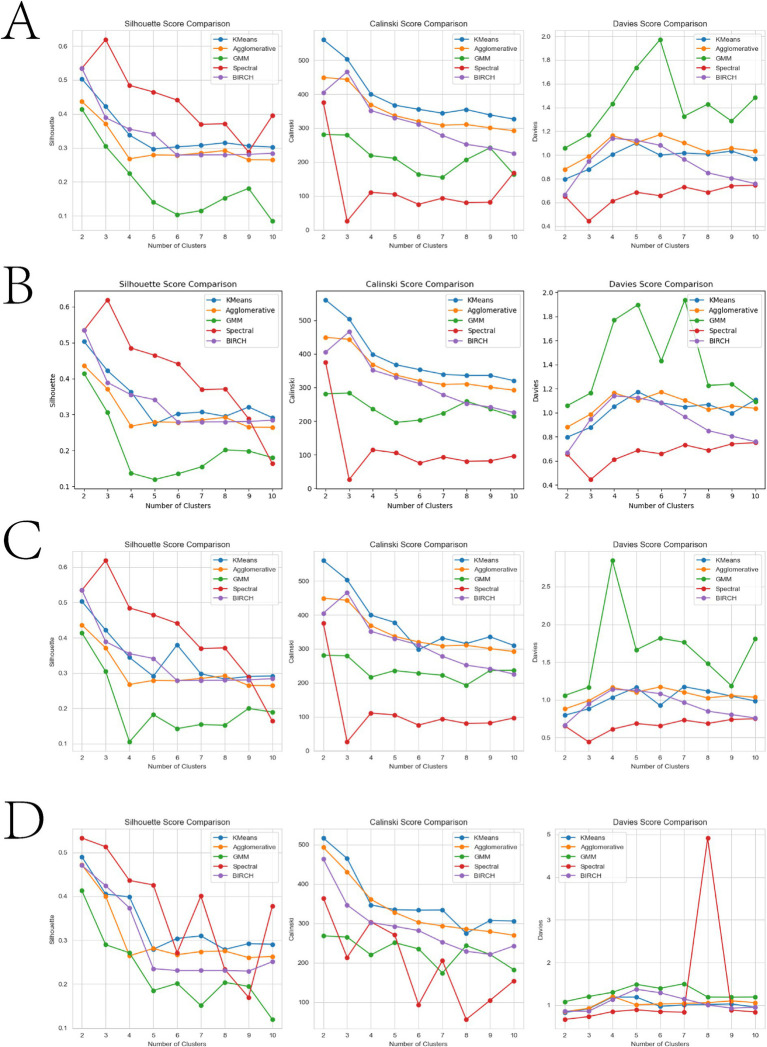
Performance evaluation of different algorithms for the identification of MHD subtypes. Panels **(A–D)** illustrate the performance evaluation results of various clustering algorithms applied to the features selected by the integrated machine learning algorithm based on four body composition and metabolic indices related to physical function (BMR, SMM, FFM, and PBF) for the identification of MHD subtypes. Each subplot **(A–D)** corresponds to one body composition and metabolic indices related to physical function, assessed by the silhouette coefficient, Calinski-Harabasz index and Davies-Bouldin score across five algorithms (K-Means, hierarchical clustering, GMM, spectral clustering and BIRCH).

In this study, we conducted exploratory clustering analyses using features related to four body composition and metabolic indices related to physical function (BMR, SMM, FFM, and PBF) to identify body-composition-based patient patterns within the MHD cohort. Five algorithms were evaluated, including K-Means, Agglomerative Clustering, Gaussian Mixture Model (GMM), Spectral Clustering, and BIRCH. Because the analysis was cross-sectional and not linked to clinical outcomes, the resulting groups were interpreted as exploratory body-composition clusters rather than validated clinical subtypes. The performance evaluation results (Figure 4) showed that different algorithms captured different aspects of cluster structure. Agglomerative clustering and GMM generally showed better inter-cluster separation according to the Calinski-Harabasz index and silhouette coefficient, whereas BIRCH showed relatively good intra-cluster compactness according to the Davies-Bouldin index. However, clinical interpretability also depended on whether the resulting clusters were balanced and whether they differed meaningfully in SMM, FFM, PBF, and BMR. For example, in the BMR indicator, hierarchical clustering achieved a silhouette coefficient of 0.58, and GMM reached a Calinski-Harabasz index of 350, indicating that these two algorithms could effectively distinguish MHD patient subtypes based on BMR.

Cluster-level comparisons showed that the KMeans and Agglomerative solutions provided the most clinically interpretable two-cluster patterns (Table 3). In the KMeans solution, Cluster 0 included 293 patients (65.5%) and was characterized by lower standardized values of TBW, FFM, SMM, and BMR, together with a higher PBF value. In contrast, Cluster 1 included 154 patients (34.5%) and showed higher TBW, FFM, SMM, and BMR, with lower PBF. Specifically, standardized SMM was lower in Cluster 0 than in Cluster 1 (−0.61 ± 0.43 vs. 1.16 ± 0.72, *p* < 0.001), as were FFM (−0.61 ± 0.42 vs. 1.16 ± 0.71, *p* < 0.001), TBW (−0.61 ± 0.43 vs. 1.16 ± 0.71, *p* < 0.001), and BMR (−0.61 ± 0.42 vs. 1.16 ± 0.71, *p* < 0.001). PBF showed the opposite pattern, being higher in Cluster 0 than in Cluster 1 (0.18 ± 0.92 vs. -0.34 ± 1.05, *p* < 0.001). This pattern suggests a relatively low-muscle/higher-fat-percentage profile in Cluster 0 and a relatively preserved lean-mass profile in Cluster 1. A similar pattern was observed in the Agglomerative clustering solution. Cluster 0 included 187 patients (41.8%) and showed higher standardized TBW, FFM, SMM, and BMR, with lower PBF, whereas Cluster 1 included 260 patients (58.2%) and showed lower lean-mass and water-compartment indices with higher PBF. For example, standardized SMM was 0.94 ± 0.81 in Cluster 0 and −0.67 ± 0.39 in Cluster 1 (*p* < 0.001), while PBF was −0.22 ± 1.06 in Cluster 0 and 0.16 ± 0.92 in Cluster 1 (*p* = 0.0007). These findings support the presence of body-composition gradients rather than fully validated disease subtypes.

**Table 3:** Standardized body-composition characteristics of clinically interpretable clustering solutions.

Variable	KMeans cluster 0, *n* = 293	KMeans cluster 1, *n* = 154	*p*-value	Interpretation
TBW	−0.61 ± 0.43	1.16 ± 0.71	<0.001	Cluster 1 higher body water/lean mass
FFM	−0.61 ± 0.42	1.16 ± 0.71	<0.001	Cluster 1 higher fat-free mass
SMM	−0.61 ± 0.43	1.16 ± 0.72	<0.001	Cluster 1 higher skeletal muscle mass
BMR	−0.61 ± 0.42	1.16 ± 0.71	<0.001	Cluster 1 higher resting-metabolic estimate
PBF	0.18 ± 0.92	−0.34 ± 1.05	<0.001	Cluster 0 higher fat percentage
Minerals	−0.57 ± 0.41	1.09 ± 0.87	<0.001	Cluster 1 higher mineral-related index
Protein	−0.61 ± 0.43	1.16 ± 0.72	<0.001	Cluster 1 higher protein-related lean index

To address the clinical interpretability of the clusters and to provide an explicit between-cluster comparison, we further characterized the consolidated whole-cohort two-cluster KMeans solution in terms of demographic, anthropometric and body-composition variables expressed in their original clinical units (Table 4). This solution reproduced the lean-mass gradient described above. One cluster (Cluster A; *n* = 156, 34.9% of the cohort) comprised taller, heavier patients (mean height 174.3 cm, weight 77.4 kg, BMI 25.5 kg/m^2^) with markedly higher total body water (43.7 vs. 30.5 L), intracellular water (26.7 vs. 18.6 L), mineral content (4.1 vs. 2.9 kg) and protein-related lean indices, whereas the other cluster (Cluster B; *n* = 291, 65.1%) comprised shorter, lighter patients (height 161.4 cm, weight 57.0 kg, BMI 22.0 kg/m^2^) with correspondingly lower water, mineral and lean-mass indices. Although Cluster A had a higher absolute body fat mass (18.1 vs. 15.6 kg), its relative body fat fraction was lower (≈23% vs. ≈27% of body weight), which is consistent with the higher PBF observed in the lower-lean-mass cluster in Table 3. The extracellular-to-total-body-water ratio (ECW/TBW), an index of relative over-hydration, was comparable between the two clusters (≈0.39), indicating that the separation was driven mainly by body size and lean/fat composition rather than by fluid-overload status. All anthropometric, water-compartment, mineral and lean-mass differences were highly significant (*p* < 0.001), whereas ECW/TBW did not differ materially.

**Table 4:** Between-cluster comparison of anthropometric and body-composition characteristics for the consolidated whole-cohort two-cluster KMeans solution (silhouette = 0.39; Calinski-Harabasz = 289.8; Davies-Bouldin = 1.12), expressed in original clinical units.

Characteristic	Cluster A: higher body water/preserved lean mass (*n* = 156, 34.9%)	Cluster B: lower body water/reduced lean mass (*n* = 291, 65.1%)	*p*
Height (cm)	174.3	161.4	<0.001
Weight (kg)	77.4	57.0	<0.001
BMI (kg/m^2^)	25.5	22.0	<0.001
Total body water, TBW (L)	43.7	30.5	<0.001
Intracellular water, ICW (L)	26.7	18.6	<0.001
Extracellular water, ECW (L)	17.1	11.9	<0.001
ECW/TBW	0.39	0.39	ns
Minerals (kg)	4.1	2.9	<0.001
Body fat mass, BFM (kg)	18.1	15.6	<0.001
Body fat fraction, BFM/weight	~23%	~27%	<0.001

Values are cluster means back-transformed from standardized centroids to original units using the cohort mean and SD reported in Table 2. *p*-values refer to between-cluster comparisons (Welch *t*-test on standardized values); ns, not significant (ECW/TBW). Cluster A corresponds to the higher-lean-mass group (Cluster 1 in Table 3) and Cluster B to the lower-lean-mass group (Cluster 0 in Table 3); the small difference in cluster sizes versus Table 3 (156/291 vs. 154/293) reflects the broader body-composition feature set used for this consolidated solution.

In clinical terms, these two data-driven groups are most plausibly interpreted as a relatively preserved musculoskeletal and nutritional phenotype (larger body size; higher muscle, protein and mineral mass; lower relative adiposity) versus a low-lean-mass, relatively higher-fat-fraction phenotype that may flag patients at greater risk of sarcopenia, frailty and protein-energy wasting. We emphasize, however, that because the present dataset contained no handgrip strength, gait speed, dietary intake, inflammatory markers or serum-albumin data, and no longitudinal clinical outcomes, these labels remain descriptive body-composition phenotypes rather than formally diagnosed clinical subtypes; their prognostic and functional relevance must be tested against directly measured outcomes in future external cohorts (see Discussion).

**Figure 5:**
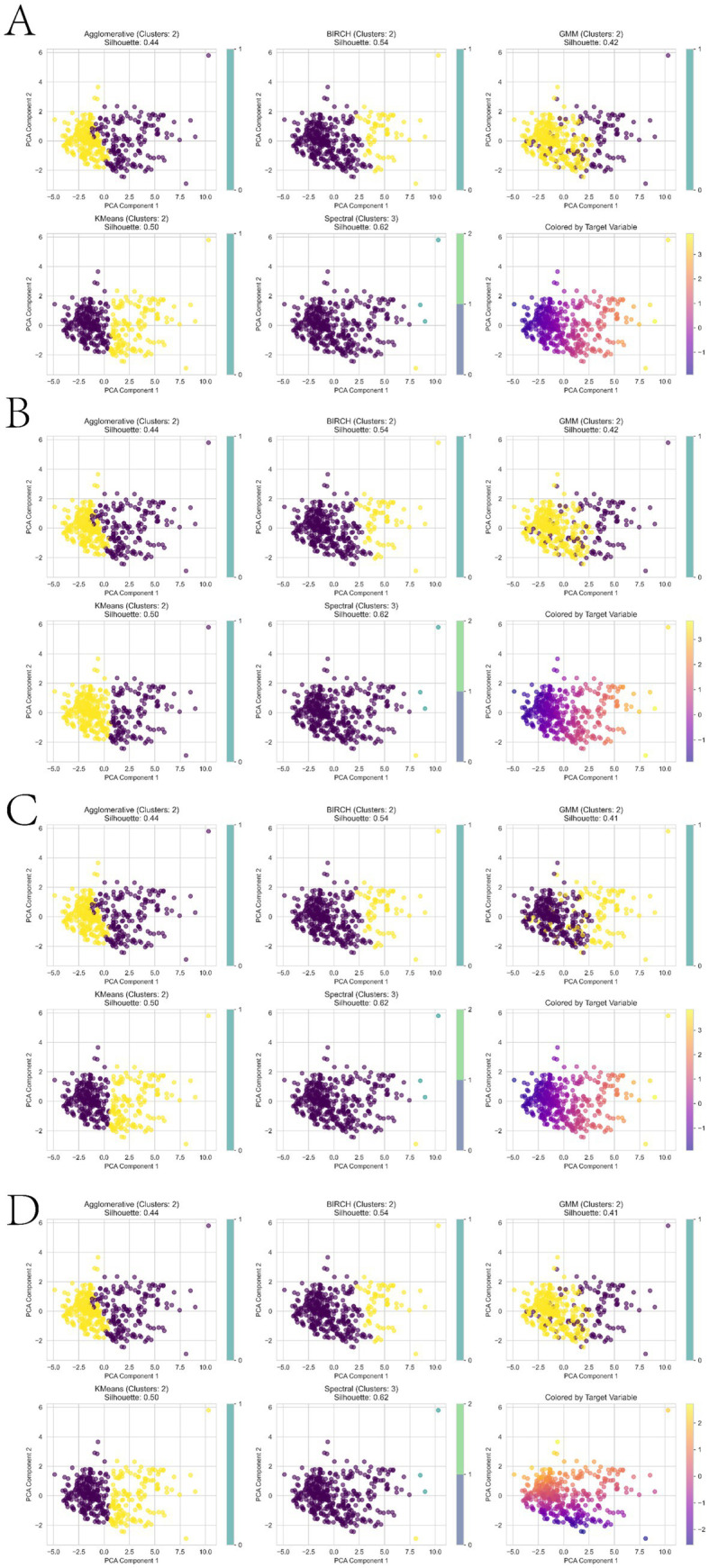
t-SNE clustering map of the optimal clustering results based on different algorithms. **(A–D)** Distribution of four physical-function-related body composition and metabolic indices (BMR, SMM, FFM and PBF). Each point is one patient; color denotes cluster membership. Within each panel, subplots 1–5 show the optimal results of hierarchical clustering, BIRCH, GMM, K-Means and spectral clustering, and subplot 6 maps the index value onto the t-SNE space.

Although Spectral clustering showed favorable values for some internal clustering metrics, its cluster distribution was highly imbalanced in the uploaded cluster summary table, with 443 patients in Cluster 0, 3 patients in Cluster 1, and 1 patient in Cluster 2. Therefore, despite some statistical separation, this solution was not considered clinically interpretable and should be viewed mainly as detecting outlying patterns rather than meaningful patient subtypes. In the FFM and PBF indicators, GMM and hierarchical clustering also showed high Calinski-Harabasz indices and silhouette coefficients (Figures 4C,D), further validating their advantages in subtype identification. Additionally, the BIRCH algorithm performed best in the Davies-Bouldin index, indicating good intra-cluster compactness. For instance, in the BMR indicator, K-Means had the lowest Davies-Bouldin index of 0.42 (Figure 4A), suggesting good intra-cluster compactness. In the SMM and PBF indicators, BIRCH had Davies-Bouldin indices of 0.40 and 0.35, respectively (Figures 4B–D), further highlighting its advantage in maintaining intra-cluster compactness. The t-SNE clustering maps (Figure 5) intuitively displayed the optimal clustering results of different algorithms. Hierarchical clustering and GMM generally distinguished different subtypes well, such as in the BMR and PBF indicators, where the clustering results of hierarchical clustering and GMM showed clear inter-cluster separation with distinct boundaries between different color regions (Figures 5A–D). K-Means and spectral clustering also showed some inter-cluster differences, but the distribution of points within clusters was relatively dispersed. The clustering results of the BIRCH algorithm exhibited more compact intra-cluster point distribution but relatively lower inter-cluster separation. The sixth subplot (the last figure in each group of Figure 5) showed that the specific values of the four indicators presented a gradient distribution in the t-SNE two-dimensional space, which corresponded to the clustering results to some extent, provided visual support for the clustering patterns observed in this dataset. However, t-SNE visualization should not be interpreted as external validation of the clusters or as evidence that the clusters represent clinically validated subtypes.

### 3.3 Correlation analysis results between target body composition and metabolic indices related to physical function and key indicators

**Figure 6:**
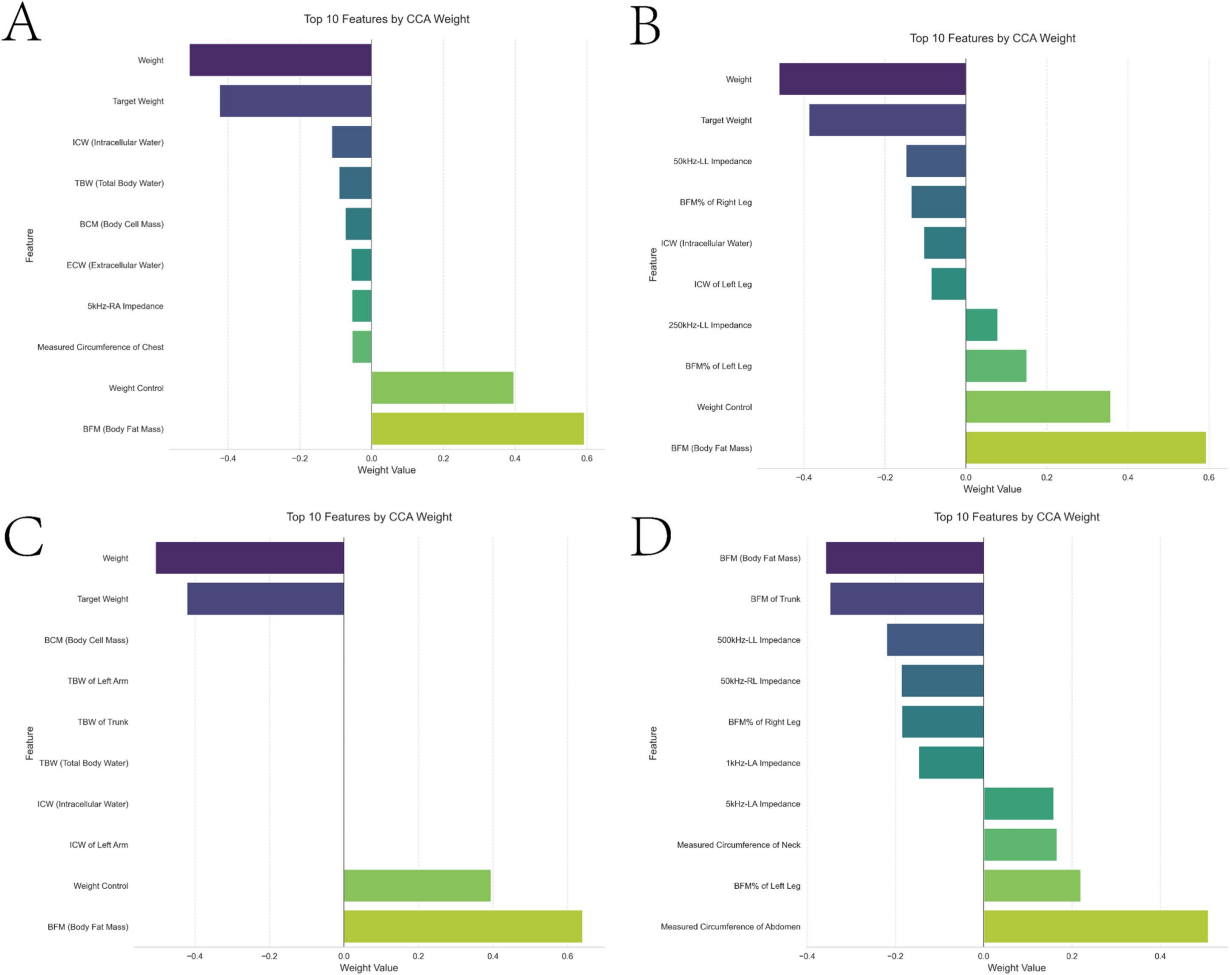
The correlation analysis results based on the CCA algorithm. **(A–D)** Correlation analysis results between four body composition and metabolic indices related to physical function and the top features selected by SHAP values. Based on the CCA weights, the top 10 features corresponding to each indicator and their weight ranges (from −0.4 to 0.9) are as follows: The features related to BMR include intracellular water (ICW), total body water (TBW), body cell mass (BCM), etc.; The features related to SMM include 50 kHz L/L impedance, right leg BFNNs, left leg ICW, etc.; The features related to FFM include body cell mass (BCM), left arm TBW, trunk TBW, etc.; The features related to PBF include retained impedance and water-distribution variables, such as 500 kHz L/L impedance and selected segmental water measures; direct fat-mass variables were excluded from the PBF model to reduce circularity. The weight values indicate the correlation strength between the features and the target variables, with positive values representing positive correlations and negative values representing negative correlations. These features mainly involve body composition indicators and impedance measurements, reflecting their statistical association with, and contribution to the model estimation of, body composition and metabolic indices related to physical function. They should not be interpreted as causal factors.

The correlation analysis results based on the CCA (Canonical Correlation Analysis) algorithm indicate that there are significant correlations between four body composition and metabolic indices related to physical function (BMR, SMM, FFM, PBF) and the top features selected through SHAP values (Figures 6A–D). Specifically, BMR showed positive model-based associations with ICW, TBW, and BCM. This pattern is consistent with the known relationship between body water, metabolically active body mass, and BIA-derived estimates of resting metabolism, but it should not be interpreted as evidence that these features causally increase BMR. SMM is correlated with features such as 50 kHz L/L impedance, right leg BFNNs, and left leg ICW. The impedance value and selected fat-distribution variables showed model-based associations with SMM, suggesting that the retained BIA-derived variables may reflect differences in tissue conductivity and body composition patterns. These associations should be interpreted descriptively rather than causally. FFM showed positive associations with BCM, left arm TBW, and trunk TBW, which is consistent with the water-rich composition of lean tissue. However, these findings indicate statistical associations within the model rather than causal determinants of FFM. PBF was associated with retained impedance and water-distribution variables, including 500 kHz L/L impedance and selected segmental water measures. Because direct fat-mass variables were excluded from the PBF model to reduce circularity, these findings should be interpreted as model-based associations among the retained BIA-derived variables rather than as causal or mechanistic relationships. Because body fat mass and segmental fat-mass terms were excluded from the PBF model to reduce circularity, these associations should be interpreted as model-based correlations among the remaining BIA-derived variables rather than direct fat-mass-to-PBF relationships.

## 4 Discussion

In this study, the SHAP model was employed to analyze the importance of various features in the regression prediction of body composition and metabolic indices related to physical function (BMR, FFM, SMM, and PBF). The results demonstrated that the SHAP model could effectively identify the most influential features for the prediction outcomes. In the analysis of BMR, ECW, TBW, and height were identified as the most important features affecting model predictions. ECW and TBW, as indicators of body water distribution, were strongly associated with BMR in the model, which is consistent with the known relationship between body water, lean mass, and BIA-derived estimates of resting metabolism (11–13). Height, as an indicator of body size, was also strongly associated with BMR, consistent with the fact that body size is incorporated into many BMR estimation frameworks. For the FFM indicator, TBW and height were the most important features, showing the highest SHAP values. ICW, minerals, body weight, and BFM also had a significant impact on model predictions. TBW and ICW, as indicators of body water distribution, are closely related to fat-free mass (FFM) because water occupies an important proportion in lean tissues such as muscles and bones (14). Height and body weight were also associated with FFM, which likely reflects the structural relationship between body size and BIA-derived lean-mass estimates. In the analysis of SMM, height and TBW were the most important features, showing the highest SHAP values. Height and TBW, as important indicators of individual body size and water distribution, are closely related to skeletal muscle mass (SMM). Minerals were also associated with SMM in the model, although this should be interpreted as a statistical association within the BIA-derived feature set rather than a causal effect (15, 16). For the PBF indicator, height and TBW were the most important features, showing the highest SHAP values. Minerals, BFM, ECW, and ICW also had a significant impact on model predictions. Height and TBW, as important indicators of individual body size and water distribution, are closely related to percentage body fat (PBF).

On the other hand, through various clustering algorithms, subtypes of MHD patients based on body composition and metabolic indices related to physical function were successfully identified. These indicators not only reflect the metabolic status and body composition distribution of patients but are also closely related to muscle health and fat storage. Hierarchical clustering and Gaussian Mixture Model (GMM) generally showed better inter-cluster separation, especially in terms of the Calinski-Harabasz index and silhouette coefficient, indicating that these two algorithms can effectively identify subtypes of MHD patients based on these biological indicators. The BIRCH algorithm performed best in the Davies-Bouldin index, showing good intra-cluster compactness. The t-SNE clustering map further intuitively demonstrated the optimal clustering results of different algorithms. Hierarchical clustering and GMM were able to distinguish different subtypes well in most indicators, with clear boundaries between different color regions. These results provide descriptive support for the presence of body-composition-based clustering patterns in this dataset. However, because the clusters were derived from cross-sectional BIA data and were not linked to clinical outcomes, they should be considered exploratory rather than validated disease subtypes. Clinically, the cluster-level characteristics provide a more concrete basis for interpreting the exploratory clustering results. In the KMeans solution, Cluster 0 showed lower standardized SMM, FFM, TBW, and BMR but higher PBF, whereas Cluster 1 showed higher SMM, FFM, TBW, and BMR with lower PBF. This pattern is compatible with a relatively low-lean-mass/higher-fat-percentage body-composition profile versus a relatively preserved lean-mass profile. When the same two-cluster solution was expressed in original clinical units (Table 4), the two groups differed substantially not only in standardized lean-mass indices but also in demographic and anthropometric terms: the preserved-lean-mass cluster was taller (174.3 vs. 161.4 cm), heavier (77.4 vs. 57.0 kg) and had higher BMI (25.5 vs. 22.0 kg/m^2^), higher total body water (43.7 vs. 30.5 L) and mineral mass (4.1 vs. 2.9 kg), yet a lower relative body fat fraction (≈23% vs. ≈27%), whereas the extracellular-to-total-body-water ratio was comparable (≈0.39), arguing against fluid overload as the main driver of separation. A similar gradient was observed in the Agglomerative clustering solution. These patterns may resemble clinically recognizable nutritional and musculoskeletal phenotypes in MHD patients, such as sarcopenic-obesity-like or protein-energy-wasting-related vulnerability. However, formal diagnosis of sarcopenia, sarcopenic obesity, or protein-energy wasting was not possible because the present dataset did not include handgrip strength, gait speed, dietary intake, inflammatory markers, serum albumin, weight-loss history, or other diagnostic criteria. Therefore, these clusters should be regarded as exploratory body-composition patterns rather than validated clinical subtypes. Future research should examine whether these exploratory clusters are reproducible in external cohorts and whether they are associated with directly measured physical function, hospitalization, falls, or mortality.

Finally, CCA combined with SHAP-selected features was used to explore statistical associations between the four body composition and metabolic indices and the retained BIA-derived variables. BMR refers to the minimal energy metabolic rate required to maintain basic vital activities in a state of wakefulness and extreme rest. In this study, BMR was positively correlated with intracellular water (ICW), total body water (TBW), and body cell mass (BCM). Intracellular water (ICW) and total body water (TBW) reflect the water balance inside and outside cells, which is the basis for maintaining cellular metabolism and physiological functions. Body cell mass (BCM) represents the number of metabolically active cells and is an important component of basal metabolism. The positive association between these features and BMR is consistent with the established physiological link between metabolically active mass, body water, and basal metabolism; however, this pattern should be interpreted as correlational rather than as evidence that these features drive BMR (17–20). Skeletal muscle mass (SMM) is a key factor in maintaining physical function and metabolic health. This study found that SMM was correlated with 50 kHz L/L impedance, right leg BFNNs, and left leg ICW. 50 kHz L/L impedance is a bioimpedance measurement that can reflect tissue conductivity and indirectly assess muscle mass. Since skeletal muscle has good conductivity, a lower impedance value is usually associated with increased muscle mass. The negative correlation between right leg BFNNs (fat mass) and SMM indicates that an increase in fat tissue may relatively reduce skeletal muscle mass. One possible interpretation, based on prior literature, is that adiposity-related inflammatory or endocrine pathways may be linked to poorer muscle status; however, this mechanism was not tested in the present study (21–23). Fat-free mass (FFM) refers to body mass without fat, mainly composed of skeletal muscle and bone. In this study, FFM was positively correlated with body cell mass (BCM), left arm TBW, and trunk TBW. The positive association between these features and FFM is consistent with the contribution of cellular mass and water content to BIA-derived lean-mass estimates, but it does not demonstrate that changing these features would increase FFM. Skeletal muscle is the largest protein reservoir in the body, and proteins play important roles in cell construction and physiological and biochemical processes. Therefore, maintaining a higher body cell mass and a reasonable distribution of water content is significant for enhancing metabolic capacity and physical performance. Percentage body fat (PBF) is an important indicator for assessing obesity and metabolic health. Because direct fat-mass and segmental fat-mass variables were excluded from the PBF model to reduce circularity, the PBF-related findings should be limited to associations with retained impedance and water-distribution variables. No causal inference regarding fat mass and PBF can be drawn from this analysis. The negative correlation between 500 kHz L/L impedance and PBF may be because high-frequency current can penetrate cell membranes, and the measurement reflects the content of total body water (TBW) and intracellular water (ICW). Therefore, the association between impedance variables and PBF may reflect differences in tissue conductivity and water distribution, but this interpretation remains descriptive and hypothesis-generating (24). Throughout this section it should be emphasized that the SHAP and CCA results quantify statistical associations within the models rather than causal effects. The biological and mechanistic explanations offered above, for example, the proposed roles of metabolically active cell mass in basal metabolism, of adipose-derived inflammatory signaling in muscle loss, and of high-frequency impedance in fat estimation, are drawn from prior literature and are presented as plausible interpretive hypotheses, not as relationships demonstrated by the present cross-sectional, single-device data.

The clinical value of these findings should be framed carefully. Because BMR, FFM, SMM, and PBF can be read directly from the same body composition analyzer, the models do not replace measurement; rather, the interpretable feature rankings indicate which routinely captured parameters most concisely summarize a patient’s musculoskeletal and metabolic status, supporting their potential use as low-burden screening signals for sarcopenia and protein-energy wasting at the bedside. A caveat specific to dialysis is fluid balance. In MHD patients, body water shifts substantially across the dialysis cycle: inter-dialytic weight gain expands extracellular water, and a single hemodialysis session can remove several liters, so ICW, ECW, and TBW, and the impedance-derived indices computed from them, depend heavily on when the measurement is taken relative to dialysis and on the patient’s volume status and residual kidney function. Consequently, the strong contribution of the water compartments to every index is physiologically expected, but it also means that associations such as that between cellular water and these indices may in part reflect transient volume state rather than stable body composition. For such signals to be clinically reliable, measurements should be standardized to a fixed point in the dialysis cycle, ideally shortly after dialysis, at or near estimated dry weight, and volume status should be explicitly taken into account when interpreting results for individual patients.

Several limitations temper these conclusions. First, the four outcomes are body composition and resting-metabolic indices rather than directly measured physical activity; although they are reasonable correlates of functional capacity, they cannot substitute for direct physical-activity measurement using accelerometers, pedometers, or wearable activity trackers, or for validated physical-activity questionnaires such as the International Physical Activity Questionnaire (IPAQ); none of which was available in this dataset. Second, both the predictors and the outcomes were generated by a single bioimpedance device, and several predictor-outcome relationships, for example those linking height and TBW to BMR, FFM, and SMM, are partly structural or physiologically deterministic. This shared measurement origin and built-in dependency inflate apparent performance and largely explain the near-perfect *R*^2^ values; they should therefore not be read as evidence of clinically novel prediction. We explicitly acknowledge that, because FFM, SMM, BMR, and PBF are themselves computed by the device from body size, water compartments, and the same raw resistance/reactance signals, the regression models partly reconstruct relationships already embedded in the manufacturer’s algorithms rather than discovering novel ones; the near-perfect *R*^2^ values should be read in exactly this light. The regression component is therefore best understood as a transparency and internal-consistency demonstration, identifying which routinely reported parameters most compactly summarize each index, rather than as independent prediction. Two analyses are needed to disentangle genuine association from device-internal reconstruction and are planned for future work: a leakage-quantifying sensitivity analysis that progressively removes the most structurally related water-compartment and body-size predictors and reports the resulting decline in *R*^2^, and external validation of each index against an independent, non-BIA reference standard. Third, the candidate features were limited to demographics and bioimpedance output, omitting laboratory data, dialysis adequacy and prescription, comorbidity burden, medications, diet, and lifestyle, factors that strongly shape physical function in the real world—which likely limits external validity. Fourth, the strict inclusion criteria (age 18–65 years, restricted BMI and body-fat ranges, and exclusion of major comorbidities such as diabetes and cardiovascular disease) produced a relatively healthy and homogeneous sample that is not representative of the broader, frequently older and multi-morbid, dialysis population. This point deserves particular emphasis: diabetes and cardiovascular disease are among the most prevalent comorbidities in dialysis patients, diabetic nephropathy is a leading cause of ESRD and cardiovascular disease is the leading cause of death in MHD patients, so their exclusion, although adopted to reduce confounding and obtain a homogeneous modeling sample, markedly narrows the applicability of our findings to the real-world MHD population. Accordingly, the present models should be regarded as derived in a selected, comparatively healthy subgroup, and they must be re-derived and validated in unselected, multi-morbid MHD cohorts, explicitly including patients with diabetes and cardiovascular disease, before any clinical use is considered. Finally, no external validation was performed. Model assessment was limited to an internal 80:20 train-test split and 5-fold cross-validation. External validation in an independent cohort, preferably multicenter, prospective, and including unselected patients with common comorbidities, is required before clinical application In addition, the timing of BIA measurement relative to the dialysis session was not standardized or incorporated into the analysis, which may have influenced ICW, ECW, TBW, and the indices derived from them.

Finally, CCA combined with SHAP-selected features was used to explore model-based associations between the four body composition and metabolic indices and the retained BIA-derived variables. BMR showed positive associations with ICW, TBW, and BCM, a pattern consistent with the known relationship between body water, metabolically active mass, and BIA-derived estimates of resting metabolism. However, this finding should not be interpreted as evidence that these variables causally increase BMR. SMM was associated with impedance and selected segmental water or fat-distribution variables, which may reflect differences in tissue conductivity and body composition patterns. Similarly, FFM was associated with BCM and segmental water measures, consistent with the water-rich composition of lean tissue. For PBF, because direct fat-mass and segmental fat-mass variables were excluded to reduce circularity, the observed associations should be interpreted only as correlations with the retained impedance and water-distribution variables. Overall, the SHAP and CCA analyses identify variables that contribute to model predictions or are statistically associated with the target indices within this dataset; they do not establish biological causation. Any mechanistic explanations should therefore be regarded as hypotheses supported by prior literature rather than conclusions demonstrated by the present cross-sectional study.

## 5 Conclusion

This study shows that ensemble learning models, particularly LGBM, RF, and ET, can describe the internal structure of four BIA-derived body composition and metabolic indices related to physical function (BMR, FFM, SMM, and PBF) in MHD patients from routine bioimpedance data with high accuracy, and that interpretable SHAP analysis consistently identifies total body water (TBW) and height as the dominant model contributors, without implying causal effects. We interpret these results cautiously: because predictors and outcomes share a single measurement source and are in part structurally related, the very high *R*^2^ values likely reflect expected physiological dependencies and shared device-derived calculations rather than independent clinical predictive power. The more useful contribution is an interpretable, low-burden body composition profile that, if externally validated and linked to clinical outcomes, may help flag MHD patients at risk of muscle wasting and impaired physical function. Realizing this potential will require measuring body composition at a standardized point in the dialysis cycle, incorporating laboratory, dialysis, and directly measured activity data, and most importantly, externally validating the models in independent, larger, more heterogeneous, multicenter and longitudinal cohorts before any clinical implementation.

## Data Availability

The original contributions presented in the study are included in the article/Supplementary material, further inquiries can be directed to the corresponding author.
