# Supplementary material for: Interpretable bioimpedance-based modeling of body composition and metabolic indices related to physical function in maintenance hemodialysis patients
Source: Front Nutr. 2026 Jul 22;13:1849618. doi: 10.3389/fnut.2026.1849618 (PMC13437490; doi:10.3389/fnut.2026.1849618)
Supplement: Supplementary file 1 [file Table_1.DOCX]

# **Supplementary Material**

**Supplementary Table S1.** Complete variable dictionary of the body-composition analyzer, with units and role in the models.

*Body composition was acquired with a multi-frequency segmental bioelectrical impedance analyzer (InBody 770; InBody Co., Ltd., Seoul, Republic of Korea). Of the 231 exported fields, 118 served as model inputs (116 continuous variables plus sex and a >65 vs ≤ 65 age indicator, one-hot encoded, giving 120 input features), 4 were prediction targets, and 109 were not used (identifier, acquisition timestamp, and per-variable normal-range limits). Lower/Upper Limit (Normal Range) entries share the unit of their parent variable.*

| **No.** | **Variable** | **Unit** | **Role in model** |
| --- | --- | --- | --- |
| 1 | ID | identifier | Not used |
| 2 | Height | cm | Input |
| 3 | Gender | M / F | Input (one-hot) |
| 4 | Age | years | Input (one-hot) |
| 5 | Test Date / Time | timestamp | Not used |
| 6 | Weight | kg | Input |
| 7 | Lower Limit (Weight Normal Range) | kg | Not used |
| 8 | Upper Limit (Weight Normal Range) | kg | Not used |
| 9 | TBW (Total Body Water) | L | Input |
| 10 | Lower Limit (TBW Normal Range) | L | Not used |
| 11 | Upper Limit (TBW Normal Range) | L | Not used |
| 12 | ICW (Intracellular Water) | L | Input |
| 13 | Lower Limit (ICW Normal Range) | L | Not used |
| 14 | Upper Limit (ICW Normal Range) | L | Not used |
| 15 | ECW (Extracellular Water) | L | Input |
| 16 | Lower Limit (ECW Normal Range) | L | Not used |
| 17 | Upper Limit (ECW Normal Range) | L | Not used |
| 18 | Protein | kg | Input |
| 19 | Lower Limit (Protein Normal Range) | kg | Not used |
| 20 | Upper Limit (Protein Normal Range) | kg | Not used |
| 21 | Minerals | kg | Input |
| 22 | Lower Limit (Minerals Normal Range) | kg | Not used |
| 23 | Upper Limit (Minerals Normal Range) | kg | Not used |
| 24 | BFM (Body Fat Mass) | kg | Input |
| 25 | Lower Limit (BFM Normal Range) | kg | Not used |
| 26 | Upper Limit (BFM Normal Range) | kg | Not used |
| 27 | SLM (Soft Lean Mass) | kg | Input |
| 28 | Lower Limit (SLM Normal Range) | kg | Not used |
| 29 | Upper Limit (SLM Normal Range) | kg | Not used |
| 30 | FFM (Fat Free Mass) | kg | Target |
| 31 | Lower Limit (FFM Normal Range) | kg | Not used |
| 32 | Upper Limit (FFM Normal Range) | kg | Not used |
| 33 | SMM (Skeletal Muscle Mass) | kg | Target |
| 34 | Lower Limit (SMM Normal Range) | kg | Not used |
| 35 | Upper Limit (SMM Normal Range) | kg | Not used |
| 36 | BMI (Body Mass Index) | kg/m² | Input |
| 37 | Lower Limit (BMI Normal Range) | kg/m² | Not used |
| 38 | Upper Limit (BMI Normal Range) | kg/m² | Not used |
| 39 | PBF (Percent Body Fat) | — | Target |
| 40 | Lower Limit (PBF Normal Range) | % | Not used |
| 41 | Upper Limit (PBF Normal Range) | % | Not used |
| 42 | FFM of Right Arm | kg | Not used |
| 43 | Lower Limit (FFM of Right Arm Normal Range) | kg | Not used |
| 44 | Upper Limit (FFM of Right Arm Normal Range) | kg | Not used |
| 45 | FFM% of Right Arm | % | Not used |
| 46 | FFM of Left Arm | kg | Not used |
| 47 | Lower Limit (FFM of Left Arm Normal Range) | kg | Not used |
| 48 | Upper Limit (FFM of Left Arm Normal Range) | kg | Not used |
| 49 | FFM% of Left Arm | % | Not used |
| 50 | FFM of Trunk | kg | Not used |
| 51 | Lower Limit (FFM of Trunk Normal Range) | kg | Not used |
| 52 | Upper Limit (FFM of Trunk Normal Range) | kg | Not used |
| 53 | FFM% of Trunk | % | Not used |
| 54 | FFM of Right Leg | kg | Not used |
| 55 | Lower Limit (FFM of Right Leg Normal Range) | kg | Not used |
| 56 | Upper Limit (FFM of Right Leg Normal Range) | kg | Not used |
| 57 | FFM% of Right Leg | % | Not used |
| 58 | FFM of Left Leg | kg | Not used |
| 59 | Lower Limit (FFM of Left Leg Normal Range) | kg | Not used |
| 60 | Upper Limit (FFM of Left Leg Normal Range) | kg | Not used |
| 61 | FFM% of Left Leg | % | Not used |
| 62 | TBW of Right Arm | L | Not used |
| 63 | Lower Limit (TBW of Right Arm Normal Range) | L | Not used |
| 64 | Upper Limit (TBW of Right Arm Normal Range) | L | Not used |
| 65 | TBW of Left Arm | L | Input |
| 66 | Lower Limit (TBW of Left Arm Normal Range) | L | Not used |
| 67 | Upper Limit (TBW of Left Arm Normal Range) | L | Not used |
| 68 | TBW of Trunk | L | Input |
| 69 | Lower Limit (TBW of Trunk Normal Range) | L | Not used |
| 70 | Upper Limit (TBW of Trunk Normal Range) | L | Not used |
| 71 | TBW of Right Leg | L | Input |
| 72 | Lower Limit (TBW of Right Leg Normal Range) | L | Not used |
| 73 | Upper Limit (TBW of Right Leg Normal Range) | L | Not used |
| 74 | TBW of Left Leg | L | Input |
| 75 | Lower Limit (TBW of Left Leg Normal Range) | L | Not used |
| 76 | Upper Limit (TBW of Left Leg Normal Range) | L | Not used |
| 77 | ICW of Right Arm | L | Input |
| 78 | Lower Limit (ICW of Right Arm Normal Range) | L | Not used |
| 79 | Upper Limit (ICW of Right Arm Normal Range) | L | Not used |
| 80 | ICW of Left Arm | L | Input |
| 81 | Lower Limit (ICW of Left Arm Normal Range) | L | Not used |
| 82 | Upper Limit (ICW of Left Arm Normal Range) | L | Not used |
| 83 | ICW of Trunk | L | Input |
| 84 | Lower Limit (ICW of Trunk Normal Range) | L | Not used |
| 85 | Upper Limit (ICW of Trunk Normal Range) | L | Not used |
| 86 | ICW of Right Leg | L | Input |
| 87 | Lower Limit (ICW of Right Leg Normal Range) | L | Not used |
| 88 | Upper Limit (ICW of Right Leg Normal Range) | L | Not used |
| 89 | ICW of Left Leg | L | Input |
| 90 | Lower Limit (ICW of Left Leg Normal Range) | L | Not used |
| 91 | Upper Limit (ICW of Left Leg Normal Range) | L | Not used |
| 92 | ECW of Right Arm | L | Input |
| 93 | Lower Limit (ECW of Right Arm Normal Range) | L | Not used |
| 94 | Upper Limit (ECW of Right Arm Normal Range) | L | Not used |
| 95 | ECW of Left Arm | L | Input |
| 96 | Lower Limit (ECW of Left Arm Normal Range) | L | Not used |
| 97 | Upper Limit (ECW of Left Arm Normal Range) | L | Not used |
| 98 | ECW of Trunk | L | Input |
| 99 | Lower Limit (ECW of Trunk Normal Range) | L | Not used |
| 100 | Upper Limit (ECW of Trunk Normal Range) | L | Not used |
| 101 | ECW of Right Leg | L | Input |
| 102 | Lower Limit (ECW of Right Leg Normal Range) | L | Not used |
| 103 | Upper Limit (ECW of Right Leg Normal Range) | L | Not used |
| 104 | ECW of Left Leg | L | Input |
| 105 | Lower Limit (ECW of Left Leg Normal Range) | L | Not used |
| 106 | Upper Limit (ECW of Left Leg Normal Range) | L | Not used |
| 107 | ECW/TBW | ratio | Input |
| 108 | ECW/TBW of Right Arm | ratio | Input |
| 109 | ECW/TBW of Left Arm | ratio | Input |
| 110 | ECW/TBW of Trunk | ratio | Input |
| 111 | ECW/TBW of Right Leg | ratio | Input |
| 112 | ECW/TBW of Left Leg | ratio | Input |
| 113 | BFM of Right Arm | kg | Input |
| 114 | BFM% of Right Arm | % | Input |
| 115 | BFM of Left Arm | kg | Input |
| 116 | BFM% of Left Arm | % | Input |
| 117 | BFM of Trunk | kg | Input |
| 118 | BFM% of Trunk | % | Input |
| 119 | BFM of Right Leg | kg | Input |
| 120 | BFM% of Right Leg | % | Input |
| 121 | BFM of Left Leg | kg | Input |
| 122 | BFM% of Left Leg | % | Input |
| 123 | InBody Score | points | Not used |
| 124 | Target Weight | kg | Input |
| 125 | Weight Control | kg | Input |
| 126 | BFM Control | kg | Input |
| 127 | FFM Control | kg | Not used |
| 128 | BMR (Basal Metabolic Rate) | kcal/day | Target |
| 129 | WHR (Waist-Hip Ratio) | ratio | Input |
| 130 | Lower Limit (WHR Normal Range) | ratio | Not used |
| 131 | Upper Limit (WHR Normal Range) | ratio | Not used |
| 132 | VFL (Visceral Fat Level) | grade | Not used |
| 133 | VFA (Visceral Fat Area) | cm² | Input |
| 134 | Obesity Degree | % | Input |
| 135 | Lower Limit (Obesity Degree Normal Range) | % | Not used |
| 136 | Upper Limit (Obesity Degree Normal Range) | % | Not used |
| 137 | BCM (Body Cell Mass) | kg | Input |
| 138 | Lower Limit (BCM Normal Range) | kg | Not used |
| 139 | Upper Limit (BCM Normal Range) | kg | Not used |
| 140 | AC (Arm Circumference) | cm | Input |
| 141 | AMC (Arm Muscle Circumference) | cm | Input |
| 142 | BMC (Bone Mineral Content) | kg | Input |
| 143 | Lower Limit (BMC Normal Range) | kg | Not used |
| 144 | Upper Limit (BMC Normal Range) | kg | Not used |
| 145 | TBW/FFM | ratio | Not used |
| 146 | FFMI (Fat Free Mass Index) | kg/m² | Not used |
| 147 | FMI (Fat Mass Index) | kg/m² | Input |
| 148 | 1kHz-RA Impedance | Ω | Input |
| 149 | 1kHz-LA Impedance | Ω | Input |
| 150 | 1kHz-TR Impedance | Ω | Input |
| 151 | 1kHz-RL Impedance | Ω | Input |
| 152 | 1kHz-LL Impedance | Ω | Input |
| 153 | 5kHz-RA Impedance | Ω | Input |
| 154 | 5kHz-LA Impedance | Ω | Input |
| 155 | 5kHz-TR Impedance | Ω | Input |
| 156 | 5kHz-RL Impedance | Ω | Input |
| 157 | 5kHz-LL Impedance | Ω | Input |
| 158 | 50kHz-RA Impedance | Ω | Input |
| 159 | 50kHz-LA Impedance | Ω | Input |
| 160 | 50kHz-TR Impedance | Ω | Input |
| 161 | 50kHz-RL Impedance | Ω | Input |
| 162 | 50kHz-LL Impedance | Ω | Input |
| 163 | 250kHz-RA Impedance | Ω | Input |
| 164 | 250kHz-LA Impedance | Ω | Input |
| 165 | 250kHz-TR Impedance | Ω | Input |
| 166 | 250kHz-RL Impedance | Ω | Input |
| 167 | 250kHz-LL Impedance | Ω | Input |
| 168 | 500kHz-RA Impedance | Ω | Input |
| 169 | 500kHz-LA Impedance | Ω | Input |
| 170 | 500kHz-TR Impedance | Ω | Input |
| 171 | 500kHz-RL Impedance | Ω | Input |
| 172 | 500kHz-LL Impedance | Ω | Input |
| 173 | 1000kHz-RA Impedance | Ω | Input |
| 174 | 1000kHz-LA Impedance | Ω | Input |
| 175 | 1000kHz-TR Impedance | Ω | Input |
| 176 | 1000kHz-RL Impedance | Ω | Input |
| 177 | 1000kHz-LL Impedance | Ω | Input |
| 178 | 5kHz-RA Reactance | Ω | Input |
| 179 | 5kHz-LA Reactance | Ω | Input |
| 180 | 5kHz-TR Reactance | Ω | Input |
| 181 | 5kHz-RL Reactance | Ω | Input |
| 182 | 5kHz-LL Reactance | Ω | Input |
| 183 | 50kHz-RA Reactance | Ω | Input |
| 184 | 50kHz-LA Reactance | Ω | Input |
| 185 | 50kHz-TR Reactance | Ω | Input |
| 186 | 50kHz-RL Reactance | Ω | Input |
| 187 | 50kHz-LL Reactance | Ω | Input |
| 188 | 250kHz-RA Reactance | Ω | Input |
| 189 | 250kHz-LA Reactance | Ω | Input |
| 190 | 250kHz-TR Reactance | Ω | Input |
| 191 | 250kHz-RL Reactance | Ω | Input |
| 192 | 250kHz-LL Reactance | Ω | Input |
| 193 | 50kHz-RA Phase Angle | ° | Input |
| 194 | 50kHz-LA Phase Angle | ° | Input |
| 195 | 50kHz-TR Phase Angle | ° | Input |
| 196 | 50kHz-RL Phase Angle | ° | Input |
| 197 | 50kHz-LL Phase Angle | ° | Input |
| 198 | 50kHz-Whole Body Phase Angle | ° | Input |
| 199 | Measured Circumference of Neck | cm | Input |
| 200 | Measured Circumference of Chest | cm | Input |
| 201 | Measured Circumference of Abdomen | cm | Input |
| 202 | Measured Circumference of Hip | cm | Input |
| 203 | Measured Circumference of Right Arm | cm | Input |
| 204 | Measured Circumference of Left Arm | cm | Input |
| 205 | Measured Circumference of Right Thigh | cm | Input |
| 206 | Measured Circumference of Left Thigh | cm | Input |
| 207 | Growth Score | points | Not used |
| 208 | Obesity Degree of a Child | % | Not used |
| 209 | Lower Limit (Obesity Degree of a Child Normal Range) | % | Not used |
| 210 | Upper Limit (Obesity Degree of a Child Normal Range) | % | Not used |
| 211 | Systolic | mmHg | Not used |
| 212 | Diastolic | mmHg | Not used |
| 213 | Pulse | bpm | Not used |
| 214 | Mean Artery Pressure | mmHg | Not used |
| 215 | Pulse Pressure | mmHg | Not used |
| 216 | Rate Pressure Product | mmHg·bpm | Not used |
| 217 | SMI | kg/m² | Input |
| 218 | Recommended Calorie Intake | kcal/day | Input |
| 219 | Lower Limit (BMR Normal Range) | kcal/day | Not used |
| 220 | Upper Limit (BMR Normal Range) | kcal/day | Not used |
| 221 | Systolic2 | mmHg | Not used |
| 222 | Diastolic2 | mmHg | Not used |
| 223 | Pulse2 | bpm | Not used |
| 224 | Mean Artery Pressure2 | mmHg | Not used |
| 225 | Pulse Pressure2 | mmHg | Not used |
| 226 | Rate Pressure Product2 | mmHg·bpm | Not used |
| 227 | SMM/WT | ratio | Not used |
| 228 | Z(R/Ht) | standardized | Input |
| 229 | Z(Xc/Ht) | standardized | Input |
| 230 | R/Ht | Ω·m⁻¹ | Input |
| 231 | Xc/Ht | Ω·m⁻¹ | Input |

**Supplementary Table S2.** Model hyperparameters of the stacking ensemble.

*A stacking regressor combined eight base learners through a Random-Forest meta-learner. Only the meta-learner (final_estimator) was tuned by GridSearchCV (5-fold cross-validation, scoring = R²) over n_estimators ∈ {10, 20, 50}, max_depth ∈ {5, 10, 20} and min_samples_split ∈ {2, 5, 10}; the eight base learners used fixed configurations.*

**Part A. Meta-learner (Random-Forest final_estimator) hyperparameters selected by GridSearchCV, by target.**

| **Target index** | **n_estimators** | **max_depth** | **min_samples_split** |
| --- | --- | --- | --- |
| BMR | 50 | 20 | 2 |
| SMM | 50 | 5 | 2 |
| FFM | 50 | 10 | 2 |
| PBF | 20 | 5 | 5 |

*All meta-learners additionally used random_state = 42; remaining parameters at scikit-learn defaults.*

**Part B. Fixed configuration of the eight base learners (identical for every target).**

| **Base learner** | **Configuration (fixed; not tuned)** |
| --- | --- |
| Linear Regression (lr) | scikit-learn defaults |
| Random Forest (rf, base) | n_estimators = 10; random_state = 42; others default |
| SVR (svr) | kernel = linear; C = 1.0; epsilon = 0.1 (defaults) |
| Gradient Boosting (gbr) | n_estimators = 100; learning_rate = 0.1; max_depth = 3; random_state = 42 |
| AdaBoost (ada) | n_estimators = 100; learning_rate = 1.0; random_state = 42 |
| KNN (knn) | n_neighbors = 5; weights = uniform (defaults) |
| Extra Trees (et) | n_estimators = 100; random_state = 42; others default |
| LightGBM (lgbm) | n_estimators = 100; learning_rate = 0.1; num_leaves = 31; max_depth = -1 (no limit); random_state = 42 |

**Software environment.** Python 3.10.20; scikit-learn 1.7.2; LightGBM 4.6.0; pandas 2.3.3; NumPy 2.2.6; SHAP 0.42.0; Matplotlib 3.10.9.
